# Frailty in primary care: a review of its conceptualization and implications for practice

**DOI:** 10.1186/1741-7015-10-4

**Published:** 2012-01-11

**Authors:** Alethea Lacas, Kenneth Rockwood

**Affiliations:** 1 Department of Family Medicine, Dalhousie University, Halifax, NS, Canada; 2 Department of Medicine (Division of Geriatric Medicine) Dalhousie University, Halifax, NS, Canada

**Keywords:** frailty, primary care, frailty index, comprehensive geriatric assessment

## Abstract

Frail, older patients pose a challenge to the primary care physician who may often feel overwhelmed by their complex presentation and tenuous health status. At the same time, family physicians are ideally suited to incorporate the concept of frailty into their practice. They have the propensity and skill set that lends itself to patient-centred care, taking into account the individual subtleties of the patient's health within their social context. Tools to identify frailty in the primary care setting are still in the preliminary stages of development. Even so, some practical measures can be taken to recognize frailty in clinical practice and begin to address how its recognition may impact clinical care. This review seeks to address how frailty is recognised and managed, especially in the realm of primary care.

## Background

Thousands of times each day, physicians around the world encounter older adults with multiple, interacting, medical and social problems. Often, both they and their patients can feel overwhelmed, and in an effort to start somewhere, many focus on the illness which seems most important, or the one with which they feel the most comfortable. Many times they continue despite confusion and difficulties, but even the most determined physicians can share with their less tenacious colleagues a feeling of frustration with the incompleteness of what they offer in the face of so many needs. In this brief review, we propose to cast this problem as one of the complexity of frailty.

Our goals in this review of how to recognize and address frailty in primary care are to show how frailty can be conceptualized in relation to complexity, and how principles having to do with the management of complex systems that are close to failure can be employed in everyday office practice to achieve better care for frail older adults. Specifically, we propose to look at what defines the "at risk" state that is frailty. We will then address how recognizing frailty helps us in managing older people who are frail. We also aim to call attention to the reality that defining the prospect of successful treatment requires understanding patients' problems and life goals. In this, family physicians have a natural advantage, given their training and predisposition towards thinking about not just diseases, but the patients in whom they occur. We hope to show how much of the intuition associated with this predisposition and training can be leveraged in understanding frailty and its determinants.

Complexity is a term that often is used synonymously with frailty, or in some settings, with the idea of physical frailty only [1]. Complexity has also been used in a restricted sense - and not without controversy [2] - to measure degrees of interaction in a system [3], or to describe non-linear increases in vulnerability in relation to the number of physiological systems showing dysregulation in an individual [4]. As detailed elsewhere, in general, a system is distinguished from a collection of elements in a set by virtue of the relationships between the elements having properties that depend on each other and change as a consequence of their interaction [5]. For example, grains of sand in a thimble appear to show no interaction compared with a similar volume of computer chips, which would have less interaction than a similar volume made up of living organisms, such as a snail or even algae... This definition shows the dependence of a system on the frame of reference. For example, the temperature of grains of sand in a thimble would depend on the interaction of the grains, their heat transfer, proximity to the sides of the container in relation to the temperature of the surrounding environment, and so on. For our purposes, the complexity of frailty is meant to convey the idea that, compared with single system disorders in otherwise well people, management of older adults with multi-system dysregulation must employ principles of complex systems management, such as recognition of interconnectedness and feedback (not additivity), indirect and delayed effects (making root cause analysis difficult), impossibility of implementing interventions with only one effect, and results that cannot be predicted from separate actions [6]. In a theoretical context, aging itself is a manifestation of complexity, being a multiply-determined phenomenon (although mathematical modelling can be employed to quantitate the extent of interactions in aging, even at the cellular level [7]). Clinically, prescribing drugs is a common example, where drug choice and dose adjustment depends not just on "renal dosing", for example, but on considering interactions, total number of drugs already in place, ability of the patient to achieve adherence, cost (including opportunity cost in relation to time spent with dispensing) and so on. A fundamental part of the challenge now faced in the management of older adults with multiple, interacting medical and social problems is the need not just to adapt routines of care, including clinical practice guidelines developed for single illnesses, but to change a way of thinking which views single system illness as normative.

### Development of the concept of frailty

Frailty is widely understood to represent variable vulnerability to adverse health outcomes of people of the same chronological age. In clinical medicine, it has its origins in aiming to differentiate health care needs of older adults, so as to show that "the elderly" are not homogenous. In this sense of understanding differential vulnerability, the clinical term frailty shares an important element with the statistical definition of frailty. In the statistical literature, frailty quantifies the variability to adverse outcomes (typically death) as a random effect that differs between people but is constant for any individual. Credit for primacy is given to Vaupel and colleagues for using the term frailty in this way in 1979 [8]. As is typical in science, the idea built on related constructs, such as Beard's "longevity factor", which came from actuarial literature of some decades prior to that [9]. As Beard conceived of it, the longevity factor characterized individuals throughout their life course. A low longevity factor value meant that people aged slowly; in actuarial terms; this was meant that their chance of dying increased more slowly than it did in people with a high longevity factor value. Used in this way, although the risk of death increased with age, it was constant for a particular individual. By contrast, in the clinical literature, frailty is considered to be different from this in two ways. First, it is seen to be an attribute which can change for given individuals, both increasing and decreasing. Second, we use clinical data, not actuarial data, and consider more outcomes than mortality. In normal clinical use, frailty reflects the variable vulnerability to adverse health outcomes of people of the same chronological age.

It is worth recalling that in the 1980s the idea of refining the profiles of various fitness and frailty states was a common focus in academic geriatric medicine [10]. As the discipline came of age at the same time as controlled clinical trials were maturing, geriatricians sought to define which older people were best suited to clinical trials of specialized geriatric interventions. Then, as now, the hallmark of the specialized geriatric intervention is comprehensive geriatric assessment (CGA). A CGA is the process by which systematic account is taken of the multiple, interacting medical and social needs of older adults, of how illness impacts on function, of how functional dependence affects caregivers, and of how each of these factors impact on the outcomes of health care. It is worth recalling too that CGA has two parts: both the identification of all these factors, and their management. The CGA process is not simply the enumeration of problems, but is also a management plan to address them. The particular concern in the era of controlled clinical trials of CGA was that this time-consuming approach should be targeted to those patients who were most likely to benefit. Today, that geriatric medicine should target frail older adults is a formula widely accepted [11-14], but this view did not arise spontaneously, and even now, to the extent that it might be seen to focus too much on the outcomes and frailty and, thereby, unintentionally undermine the concerns of preventing frailty, it is not entirely without controversy.

Even so, if we begin by claiming that geriatric medicine should focus on older adults who are frail, we must then ask: "Who are the frail?" We must also recognize that this focus on the frail by geriatricians cannot be a proprietary claim; there are far too many frail older adults and far too few geriatricians. Given that frailty is increasingly understood as a common stage of the life course [15], then it is obvious that it will fall to family medicine and other primary care physicians to provide the bulk of this care. With the importance of family caregivers in the lives of people, who are dependent or at risk for dependence, this review is directed towards family physicians.

### Frailty and its recognition in clinical practice

#### Frailty phenotype

Although the operational definition of frailty is widely recognized as being as yet unsettled [16-20], in general, two approaches predominate. The most widely cited is the frailty phenotype [21], in which in any individual frailty can be recognized by the presence of at least three of five particular deficits, specified as: measured slow walking speed, measured impaired grip strength, self-reports of declining activity levels, exhaustion and unintended weight loss (this last can also be measured if longitudinal data are available). People with three or more of these deficits are said to be frail and those with none are said to be robust. The term "pre-frail" is used when only one or two of these deficits is present. The originators of this consensus operational definition were motivated in large measure by the need to identify a group of people who would conform to the essence of the definition of frailty (that is, who would demonstrate an increased risk of adverse health outcomes) but who would be both clinically recognizable and not otherwise definable as being disabled (on which other operational definition then extant rested [22] or as having multiple co-morbid illnesses.)

#### The frailty index and its variants

Another approach to measuring frailty is to employ a frailty index, which is a count of health deficits. A health deficit can be any clinical symptom, sign, disease, disability, or laboratory, imaging or electrodiagnostic abnormality. The rationale for counting deficits is straight forward: the more things which an individual has wrong with them, that is, the more deficits which they accumulate, the greater their risk of an adverse health outcome. In this sense, and recalling that the idea of frailty is meant to better grade the risk of adverse health outcomes amongst people of the same age, the more deficits that someone has, the frailer they are. There are notably few restrictions on what can be counted as a health deficit, even though in different studies, which used only self-report, or mostly clinical data or some combination of self-report, observer assessed and test data [23], similar estimates of prevalence and risk have been demonstrated. Usually, at least 30 items are included in a frailty index, making it useful for secondary analyses of existing databases, including electronic medical records [24].

To develop a frailty index from existing clinical records requires assessing which variables might be considered as health deficits. Some guidelines, for which items should be included, are best followed [25]. In general, however, to be included, a health deficit should be acquired, age-associated and associated with an adverse outcome. Recent commentators are right to point out that the number of deficits included in a frailty index, (usually 30 or more), is as important as the nature of any one of them [26]. For example, hypertension meets all these criteria. If green eye colour were somehow found to be associated with poor health, it would not be included, because it is neither acquired nor age associated. Another criterion is that the deficit not saturate too early, that is, that it not be present in all or most people (a reasonable criterion for saturation appears to be about 80%, that is, any deficit present in more than 80% of people simply adds a number to both the numerator and the denominator and, thereby, does not help grade risk). Often the saturation rule is context dependent. For example, in developing a frailty index to track health risk in a group of people who all live in an assisted living facility, there is little point in counting dependence in Instrumental Activities of Daily Living (IADLs), because that is a criterion for admission and so everyone in this group would have it. By contrast, if the intent was to develop a frailty index to stratify risk in people who present to an acute care hospital, then knowing IADL dependence would be clearly important, as those with such dependence would be at higher risk than those who were IADL independent [27].

Although the idea of counting deficits is simple, it gives rise to results which are neither trivial nor obvious. For example, in several studies, deficits consistently accumulated exponentially with age, at an average relative rate of about three percent per year on a log scale. This has held both in countries in the West [24], and in China, even though the mortality associated with any degree of frailty is higher in the latter than in the former [28-30]. When considering how to adapt a frailty index to work in a primary care practice, it is worth noting that this consistency of result was obtained across many different constructions of the frailty index. Briefly, reflecting availability of items, different numbers of variables (from 20 to 130) and different variables themselves were used in the different datasets. What is more, not every dataset had equivalent items; the health variables contained in each dataset notably varied in how many functional disabilities, diagnoses and medications they recorded [24]. Even so, in each dataset, increasing values of the frailty index were highly associated with an increased risk of death. When both are combined in a multivariable model, in each case, the frailty index has always better predicted mortality than has age. This approach to counting deficits as a means of distinguishing risk amongst older adults has been independently confirmed in several studies [31-36].

In general, at any given age, women on average have more deficits than do men. Even so, although their risk increases with increasing values of the frailty index, for any given frailty index value, the risk of adverse outcomes is lower for women than for men [24]. In this sense, women tolerate their deficits better than men do. The biological basis for this better tolerance of deficits by women has yet to be established, but the finding is robust, and implies a system effect. Several explanations have been proposed, including evolutionary trade-offs, such that the price of more optimal physiological functioning during youth is a lower threshold for system failure in old age [37].

Several variants of the frailty index exist. Typically, they have been shortened by employing statistical techniques (such as factor analysis) to reduce the number of variables which are considered, and often weigh some more than others to reflect their importance in prediction [38-40]. In our experience, while a weighted frailty index will offer better prediction retrospectively, if it is tested in the same cohort in which it was derived, it typically fails to generalize with these weights to other samples [41,42]. For this reason, we tend to use index measures with more variables and to not weight them. In general, reproducibility of confidence limits is also lower when fewer variables are considered [43], and it is less easy to demonstrate grades of frailty, or to use modeling techniques, which give insight into mechanisms [24]. For these reasons, we tend to favour a frailty index with multiple items. Even though it requires that more information is considered, this is not inappropriate when trying to assess what is wrong with patients who have complex illnesses. Other, shorter frailty indices also exist, such as the Study of Osteoporotic Fractures (SOF) index developed and cross validated by Ensrud and colleagues [40,44]. It measures three items (weight loss, inability to rise from a chair, and poor energy). In general, very short indices are less able to be used for evaluating complexity mathematically; also, they do not ensure that relevant data (such as which illnesses a person has, how they are treated, how they function, whether they are safe in transfers - a constellation sometimes referred to as multidimensional impairment [45]) are collected. Where other processes are in place to ensure that such information is incorporated into the types of decisions for which a frailty measure might be useful (for example, suitability for invasive or toxic procedures, need to hospitalize or institutionalize) then this may be less a concern. One caveat when using abbreviated or simplified frailty screens is that people who cannot perform performance measures should be seen as especially at risk, and not as having "missing data", which is a common practice in epidemiological studies, and can also be the case where protocols require adherence to specific measures [46].

There are notable contrasts between the frailty phenotype [21] and the frailty index [24] approaches. Whereas the frailty phenotype *highly specifies *which items should be included in defining frailty, the frailty index approach *hardly specifies *which items to include. However, the frailty phenotype and index approaches also have much in common. It is interesting to note that the five items used in the phenotype definition can be combined in an index (from 0 to 5). Although frailty is less commonly graded by this approach, a dose response can be demonstrated. A dose response has also been shown for the three-item SOF index, based on phenotype items [40]. Even so, using just a few items is insensitive to early stages of risk, and typically shows ceiling effects, in contrast to the submaximal limit which has been demonstrated by the frailty index measures that consist of 20 or more deficits [24]. In many situations, however, doing even a few things might well be better than leaving such items entirely unassessed. Although the three-item SOF frailty index has been validated against the five phenotypic items, clinical trials of abbreviated versus more comprehensive assessments would be helpful.

#### Does recognizing frailty improve clinical care?

Identifying frailty is the first step, but, as above, ultimately the question that must be addressed is whether recognizing frailty in primary care aids in the management of people who are frail. It is widely accepted that it is worth knowing about frailty as a means of improving care, although trials of frailty recognition versus operating without the construct are lacking [18,47-51]. Frailty has been shown to be an independent marker for worse outcomes following surgery, including postoperative complications, mortality, length of stay and discharge to care facilities [52-54]. As such, frailty has been proposed as an additional component of pre-operative risk classification and as a guide in informed decision making for patients and families. Afilalo and colleagues reviewed studies of frailty and cardiovascular disease [55]. They identified that frailty confers an increased mortality risk. They too propose that frailty could be used to better define prognosis in frail patients with cardiovascular disease. Singh and colleagues explicitly call for early identification of frailty amongst patients with cardiac disease to help tailor decision making, optimization of other comorbidities and frame discussions about prognosis and goals of care with patients and their families [56]. Frailty has also been identified as an independent marker for worse outcomes in patients discharged from the emergency department [57]. Frailty conferred an increased risk of death, hospitalization, and admission to a long term care facility in the 30 days after discharge. Extrapolating from this finding, frailty could again be a key feature, if identified in the emergency department setting, which might frame target interventions and medical decision making. Robinson and colleagues looked specifically at the healthcare cost associated with surgery and the relationship to a patient's frailty. They found a clear relationship between increased frailty and increased costs, not just at the time of hospitalization for surgery, but at six months post discharge [58]. Pulignano and colleagues identified that moderately frail patients with heart failure benefited from a targeted intervention with improved clinical outcomes and improved healthcare costs [59]. Not only does identifying frailty alter health outcomes, but it has been shown that identifying frailty in primary care allows for targeted interventions that reduce cost [60].

In the family medicine setting many medical decisions revolve around preventive health manoeuvres, such as screening tests or investigations. Identifying frailty would allow for these discussions to be more appropriately targeted to patients who might live to see benefit from screening tests [61]. Strict definitions of life expectancy often do not incorporate the increased risk of mortality conferred by frailty, thus making conversations about screening less applicable. Braithwaite *et al. *present an updated framework that incorporates frailty that can be used in clinical practice to inform decisions around screening tests [61]. Although it still requires further development, their model does point towards the benefit of incorporating frailty into clinical decision making for frail patients. Ultimately clinicians may be left with a roster of screening tests that may confer benefit to their frail older patients, and a more defined list of what is unlikely to be of benefit or even likely to cause harm. The benefits of informed decision making that arise from identifying frail patients are not only limited to preventive health discussions. Including frailty in the informed decision making for any medical intervention has the potential to provide important information for both clinician and patient and will allow more rational and informed decisions [49]. Frailty provides a framework for discussion about goals of care, including end of life care goals. It may encourage proactive planning on the part of the physician and the patient and their family due to the sense of imminent morbidity and ultimately mortality that accompanies frailty [62].

On this background, it seems reasonable to suggest that identifying frailty is helpful in clinical decision making, even if at the present we lack trials to show that this changes outcomes [63]. Identifying frailty flags increased risks for complications and mortality with invasive interventions, thus empowering the physician to have appropriate conversations about potential risks and benefits with the patient and family. It allows primary care physicians to make informed recommendations and decisions around preventive and screening interventions, and, thereby, has the potential to decrease unnecessary or harmful medical testing. It provides a framework for conversations around end of life care and goals of care. It also provides a language and framework that primary care physicians can use to describe challenging complex patients who present in variable clinical states, but who are all in the common stage known as frailty. Unfortunately, integrating the identification of frailty and use of frailty as a diagnostic category has been minimally developed in family medicine.

### Concept of frailty in primary care

A review of the literature reveals that frailty in primary care is a topic in its infancy. Pub Med was systematically searched for articles with key words "primary care" or "family medicine" and "frailty". Search results were limited to the English language, adults over age 65, and articles published since 1990. With these requirements, as of 4 October 2011, 45 papers fit the criteria. The abstracts of the articles returned on the search were reviewed for relevance to family medicine or primary care. Specifically, articles were chosen if the study was in a primary care setting, the interventions were performed by a primary care provider and the patient population was a broad frail patient group, rather than a specific subtype of frail patient with targeted diseases. Any of the articles with high relevance were then reviewed and any key references that were relevant to family medicine or primary care and frailty were examined. While De Lepeleire's "Frailty: an emerging concept for general practice" directly addresses the potential for frailty as a concept to be utilized in primary care, for the most part frailty and its application as a concept in family medicine are minimally developed [51].

Many of the studies that fall under "primary care" and "frailty" utilize or test screening tools that are not directly applicable to a family physician's office or that are still in the preliminary phases of development. Many are in the phase of identifying which factors could be used to identify frailty, but do not actually test the application in primary care [64-66]. Other articles extrapolate from research conditions and propose the feasibility in a family medicine setting. A recent report using the Edmonton Frail Scale [67] is one such example [68]. Many studies use various methods to screen for frailty including questionnaires and established frailty criteria, as a way to identify groups of patients for further interventions [66,69]. The applicability of these screening techniques in a family physician's office largely has not been studied except by Cavazzini and colleagues who tested the hypothesis that primary care physicians can easily screen for poor lower extremity function in the daily routine of their clinics [70]. They concluded from their small sample of 23 physicians that this was feasible. This study was expanded on in the Frasi trial in which 39 primary care physicians used the Short Physical Performance Battery, previously tested by Cavazzini, to identify frail older patients who were in for a routine visit to their primary care doctor [71]. The *SHARE Fraily Index *is another tool which is meant to facilitate screening for frailty in primary care [72]. A web-based calculator was developed that would determine a frailty class when a primary care physician inputs five particular measurements pertaining to their patient. Like many others this tool was developed in the research realm and not tested in a primary care clinic setting for acceptability and ease of use. Other groups have tried to use alternate markers for identifying frail patients. Maly *et al. *have published a preliminary report that tried to identify what screening questions could be used to better identify which frail older people in the outpatient setting would benefit from further Comprehensive Geriatric Assessment [73]. They hypothesized that abnormal results on validated screening measures for geriatric syndromes might serve as a proxy for frailty. While they recognize that further work is needed to clarify these questions, if screening questions were proven to be surrogate markers they might be feasibly incorporated in primary care settings.

No clear winner emerges from the slim field of studies that test the accessibility and applicability of screening tests for frailty in family medicine [74]. Certainly, as many of the authors have stated, work in the field of frailty in primary care, and more specifically what tools to use to allow family physicians to quickly and reliably identify their frail older patients, is just beginning. More studies are needed to explicitly look at the feasibility of screening tools in the family medicine setting and even more broadly speaking, how to encourage the adoption of the concept of frailty by family physicians and then, how to allow it to inform management. Even so, it is important to note that a lot of research, touching essentially the same questions in primary care [75-79], might be neglected, only to be rediscovered - if not outright lost - in "new" research that screens for frailty. If frailty tools are harder to implement in primary care, then there will be no net benefit. On the other hand, to the extent that current instruments might duplicate what is essential to know in evaluating fitness and frailty, and that the range might adequately be covered by a single comprehensive assessment, this may be less a concern than might be imagined. Clearly, studies comparing multi-part, hierarchical staged tools would be useful. In general, the concept of high order functions, which integrate across many bodily systems, such as mobility, balance, function, attention, self-efficacy and social interaction (these are sometimes referred to as "state variables" [24]) could serve as useful means to track the overall state of health, broadly construed.

### Family Medicine as specialty has advantage to understanding frailty

Family physicians are already well poised to incorporate the concept of frailty into the care of their older patients. The philosophy of family medicine encourages a patient centered approach that takes into account individual goals of care, beliefs, preferences, social context and patients' experiences of illness. As such, the concept of frailty would allow for an expansion of these core values and skills when caring for the frail older people. It would allow family physicians to use a framework they are already comfortable using and apply it to a patient population that often feels overwhelming. By naming the complexity, that is, naming the vague feeling of concern, futility and challenge that often accompanies caring for the frail older person in a busy family physician's office, the physician can begin making clinical decisions and treatment recommendations in the context of frailty and its accompanying risks. It could also allow for more targeted interventions and avoidance of inappropriate ones. Moreover, adopting the concept of frailty in primary care has the potential to allow earlier identification of patients who are at risk and who are moving in and out of the frailty continuum. Again family medicine is well positioned to act on identifying those at risk for frailty [60]; this is an area in which further research is needed, but where little is likely to be lost by at least recognizing the complexity of their needs. Preventive medicine is a core component of primary care and family medicine, and if frailty was understood to be amendable to preventive measures it could be incorporated into the existing preventive medicine framework. This equally applies to secondary and tertiary prevention, especially in relation to the increased rate of adverse outcomes which arise when complex interventions are undertaken in people whose functional status and frailty go unrecognized [52,80]. As much of this information is readily known by primary care physicians, but typically does not make it way into preoperative assessments, there would appear to be a very useful, if often under-exploited role, for the primary care physician in the assessment of pre-operative risk, especially in patients undergoing elective procedures, including chemotherapy [81].

Family physicians are well poised to identify frail patients in their practice. Doing so will allow focused interventions and recommendations about medical management. There is good evidence that identifying frail patients will improve clinical outcomes and there is increasing evidence that it is cost effective. The final barrier of finding a method that is feasible for use in primary care, but comprehensive enough to allow non-arbitrary decisions to be made and justified requires further study. For now, addressing frailty in some way is clearly preferable to not addressing it at all. The discipline of using the result of the frailty assessment to develop pragmatic care plans would seem to favour basing a frailty assessment on enough information needed to make care better.

## Conclusion

This review has drawn to attention how the complexity of frailty is a challenge to traditional health care delivery, which often is based on a single diagnosis. In this regard, primary care medicine is better placed than many other specialties, in having a greater focus on the patient. This has also drawn to attention the critical role for primary care physicians in the management of older adults who are frail. This is not making a virtue of necessity (there being too few geriatricians) but in recognition that for many people an extended period of frailty will be an important part of their life course, which needs to be managed appropriately, and that the thoughtful focus on the person and not just their illnesses is a hallmark of proper primary care. Further study needs to occur to develop and test tools for frailty screening in the primary care setting. Once these tools are developed, frailty as a diagnostic category with the accompanying risks and poor prognosis can be incorporated into many clinical decisions and discussions. Ultimately the goal of facilitating the identification of frail patients in the primary care setting is to improve the quality of sensible patient centered care provided to frail older patients.

## Abbreviations

CGA: comprehensive geriatric assessment; IADL: Instrumental Activities of Daily Living; SOF: Study of Osteoporotic Fractures

## Competing interests

The authors declare that they have no competing interests.

Kenneth Rockwood receives career support from the Dalhousie Medical Research Foundation as the Kathryn Allen Weldon Professor of Alzheimer research. He has applied for funding to commercialize a version of the frailty index.

## Authors' contributions

The authors contributed equally to the drafting and revising of this paper and have read and approved the final manuscript.

## Pre-publication history

The pre-publication history for this paper can be accessed here:

http://www.biomedcentral.com/1741-7015/10/4/prepub
